# Clinical and Mycologic Characteristics of Emerging Mucormycosis Agent *Rhizopus homothallicus*

**DOI:** 10.3201/eid2907.221491

**Published:** 2023-07

**Authors:** Shivaprakash M. Rudramurthy, Shreya Singh, Rimjhim Kanaujia, Hansraj Chaudhary, Valliappan Muthu, Naresh Panda, Abhishek Pandey, Sheetal Thakur, Harsimran Kaur, Anup Ghosh, Ritesh Agarwal, Arunaloke Chakrabarti

**Affiliations:** Postgraduate Institute of Medical Education and Research, Chandigarh, India

**Keywords:** *Rhizopus homothallicus*, *Rhizopus arrhizus*, mucormycosis, fungi, fungal infections, COVID-19 associated mucormycosis, epidemiology, antifungal susceptibility testing, amplified fragment length polymorphism, India

## Abstract

We retrospectively reviewed consecutive cases of mucormycosis reported from a tertiary-care center in India to determine the clinical and mycologic characteristics of emerging *Rhizopus homothallicus* fungus. The objectives were ascertaining the proportion of *R. homothallicus* infection and the 30-day mortality rate in rhino-orbital mucormycosis attributable to *R. homothallicus* compared with *R. arrhizus.* *R. homothallicus* accounted for 43 (6.8%) of the 631 cases of mucormycosis. *R. homothallicus* infection was independently associated with better survival (odds ratio [OR] 0.08 [95% CI 0.02–0.36]; p = 0.001) than for *R. arrhizus* infection (4/41 [9.8%] vs. 104/266 [39.1%]) after adjusting for age, intracranial involvement, and surgery. We also performed antifungal-susceptibility testing, which indicated a low range of MICs for *R. homothallicus* against the commonly used antifungals (amphotericin B [0.03–16], itraconazole [0.03–16], posaconazole [0.03–8], and isavuconazole [0.03–16]). 18S gene sequencing and amplified length polymorphism analysis revealed distinct clustering of *R. homothallicus*.

Mucormycosis is an angioinvasive disease caused by the saprophytic fungi of the order Mucorales. The estimated prevalence of mucormycosis is ≈70 times higher in India than elsewhere (*1*). *Rhizopus arrhizus* is the most common etiologic agent of mucormycosis in India and worldwide (*2*). Other reported Mucorales include *Apophysomyces variabilis*, *Cunnighamella* spp*.*, *Lichtheimia* spp., *Mucor* spp., *Rhizomucor* spp., *Rhizopus microsporus*, *Rhizopus homothallicus*, *Saksenaea vasiformis*, *Syncephalastrum* spp., and *Thamostylum lucnowense* (*3*–*7*). *R. arrhizus* was the most common causative agent even during the recent outbreak of COVID-19–associated mucormycosis (CAM) in India (*8*). Although infection with *R. homothallicus* was also reported in a few patients (*9*), the importance of mucormycosis caused by *R. homothallicus* is unclear. We report the percentage of patients with mucormycosis caused by *R. homothallicus* at our center (Postgraduate Institute of Medical Education and Research [PGIMER], Chandigarh, India) and describe clinical features, mycologic characteristics, antifungal susceptibility, treatment, and mortality rates. We also assess whether the mortality rate from rhino-orbital mucormycosis (ROM) caused by *R. homothallicus* is different from that of *R. arrhizus* disease.

The primary objectives of this study were to assess the proportion of patients with mucormycosis caused by *R. homothallicus* and 30-day mortality rate from ROM caused by *R. homothallicus* and to determine whether the species of Mucorales (*R. homothallicus* vs. *R. arrhizus*) was an independent predictor of death from ROM. The secondary objectives were to compare the profile of patients infected with *R. homothallicus* versus *R. arrhizus* and to ascertain the mycologic characteristics of *R. homothallicus* isolates by conducting antifungal-susceptibility testing (AFST) and amplified fragment length polymorphism (AFLP) analysis.

## Methods

### Study Design and Setting

We performed a retrospective study at PGIMER on a 10-month period (January–October 2021). Our center’s institutional ethics committee approved the study protocol. We were granted a consent waiver because the study was a retrospective analysis of anonymized patient data. We report the study according to the Strengthening the Reporting of Observational Studies in Epidemiology statement (*10*). Data for a few participants published in this study have been reported in previous studies (*11*–*13*). We conducted the study in accordance with Declaration of Helsinki guidelines; the study was approved by the PGIMER Institutional Review Board (approval no. PGI/IEC/2021/001101) in August 2021.

### Study Participants

We enrolled consecutive patients with ROM caused by *R. homothallicus* and *R. arrhizus.* The patients in whom mucormycosis caused by *R. homothallicus* and *R. arrhizus* was diagnosed were identified from our mycology laboratory records; we collected relevant clinical data from the patient records. We followed the study participants until discharge or 30 days after their mucormycosis was diagnosed. We sought any missing information for the study by contacting patients by telephone. We excluded patients for whom information was not adequate or not available. We obtained informed consent from all patients involved in the study.

### Data Collection and Variables

For eligible patients with ROM, we retrieved data on age, sex, and risk factors for mucormycosis (e.g., diabetes mellitus, COVID-19 infection, organ transplantation, immunosuppressive therapy, hematologic malignancies). We defined diabetes mellitus as recently diagnosed if the disease was detected (hyperglycemia and glycated hemoglobin >6.5%) during the current illness. We also retrieved clinical details, including signs and symptoms of ROM, treatment, and outcome of all patients (noted on follow up at 30 days after mucormycosis diagnosis, irrespective of discharge from hospital). All the study participants received the standard of care treatment in accordance with our institutional protocol.

### Study Definitions

Mucormycosis was diagnosed in patients with compatible clinical and radiologic features and confirmed by histopathologic or microbiologic methods, as previously described (*8*). We arbitrarily defined COVID-19–associated mucormycosis as mucormycosis diagnosed simultaneously with or within 3 months of virologically confirmed COVID-19 (*14*).

### Phenotypic Identification of the Isolates

**Figure 1:**
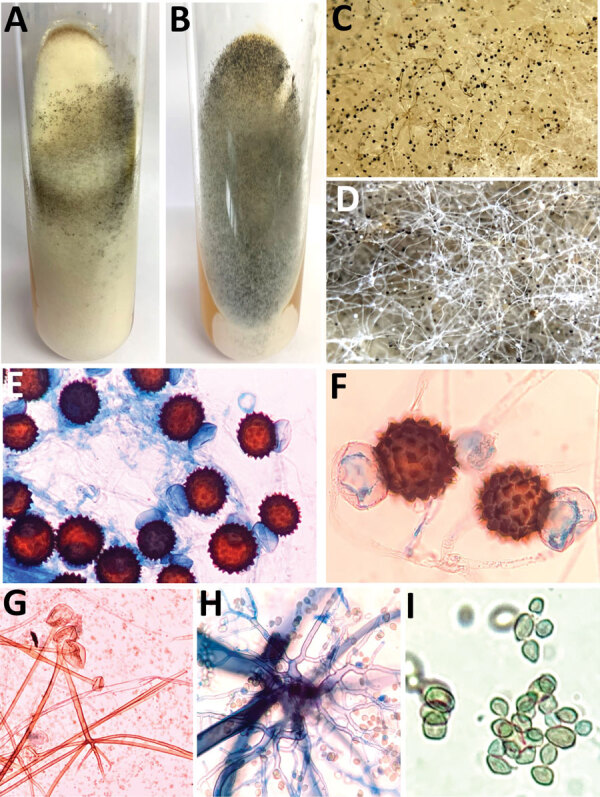
Macroscopic and microscopic characteristics of *Rhizopus arrhizus* and *R. homothallicus* isolated from tissue samples from patients enrolled in a 10-month retrospective study, Chandigarh, India, January–October 2021. A, B) Macroscopic appearance of colonies of *R. homothallicus* (A) and *R. arrhizus* (B) fungi. C, D) Macro lens image of the colonies showing dark-brown specks in *R. homothallicus* (C) and black and white dots (salt and pepper appearance) in *R. arrhizus* (D). E) Photomicrograph from a lactophenol cotton blue mount of *R. homothallicus* showing multiple reddish brown ornamented zygospores (original magnification ×400). F) Magnified image of E showing unequal suspenser cells and zygospore with prominent spinous projections. G) *R. arrhizus* showing long, unbranched sporangiophore with nodal rhizoids (original magnification ×200). H) Magnified image of extensively branched rhizoid seen in *R. arrhizus*. I) Magnified image of sporangiospores of *R. arrhizus*.

We inoculated tissue samples collected from patients on Sabouraud dextrose agar and dichloran rose bengal chloramphenicol (both from HiMedia, India) with benomyl. We isolated the Mucorales from the culture and confirmed them by demonstrating broad aseptate ribbon-shaped hyphae on direct microscopic examination of the samples. We performed phenotypic identification on the basis of the colony morphology (e.g., texture, growth rate, and color) and microscopic findings (i.e., lactophenol cotton blue mount prepared from slide culture) (*15*). We then summarized the distinctive features of *R. arrhizus* and *R. homothallicus* (Table 1; Figure 1).

**Table 1:** Mycologic characteristics of *Rhizopus arrhizus* and *R. homothallicus* based on macroscopic and microscopic findings of tissue samples from patients enrolled in a 10-month retrospective study, Chandigarh, India, January–October 2021*

Characteristic	*R. homothallicus*	*R. arrhizus*
Macroscopic appearance		
Growth rate	Fast-growing colonies and sporulation relatively slower than *R. arrhizus*	Fast-growing colonies and sporulation relatively faster than *R. homothallicus*
Obverse surface growth	Cottony, white colonies that turn **grayish to olive brown** with a variegated appearance; because of the large-sized zygospores, brownish tufts can be seen unevenly distributed throughout the mycelial growth	Cottony, white colonies that turn gray with **typical salt and pepper appearance**
Reverse macroscopic findings	Reverse surface has no pigment	Reverse surface has no pigment
Microscopic findings		
Hyphae	Aseptate hyphae, less prominent rhizoids; smooth and thick-walled intercalary chlamydospore	Aseptate hyphae, well-developed nodal rhizoids with occasional intercalary chlamydospores
Sporangiophore	Erect, unbranched, or dichotomously branched (length 300 – 2000 µm, width 5–30 µm)	Single or tufts of mostly unbranched sporangiophores (length 1000–2000 µm, width 7–18 µm)
Sporangium	Sparsely noted in cultures; when present, appears spherical and greyish brown (20– 140 µm) with conspicuous dark apophysis and subspherical columella	Spherical sporangia (20–250 µm) with short apophysis and spherical columella occupying 50% of sporangium
Sporangiospores	Spherical to broadly ellipsoidal (3–5 × 4–8 µm), hyaline, and thick-walled with less prominent striations	Lemon-shaped or subspherical to ellipsoidal (6–8 × 4.5–5 µm) with striations, rough surface
Zygospores	Homothallic†; **abundant golden brown zygospores** (60–100 µm) with stellate spinous projections, attached to large globose suspensor cells that are unequal in size	Heterothallic **zygospores not seen on primary culture**; red-brown spherical or laterally flattened (60–140 µm) with flat projections; suspensors are unequal, spherical to conical

*Key features highlighted in bold. †Differentiation from other homothallic *Rhizopus* species that produce abundant zygospores (e.g., *R. sexualis* and *R. azygosporus*) is done based on size of the suspensor cells of the zygospore and by molecular identification using the internal transcribed spacer and 28S sequences of rDNA.

### Molecular Identification of the Isolates

We confirmed identification of all *R. homothallicus* isolates from ROM cases and 6 additional *R. homothallicus* isolates from pulmonary mucormycosis cases by using a molecular sequencing method. In addition, we used sequences of 2 environmental isolates of *R. homothallicus* for comparison with clinical strains. We submitted to GenBank and included for phylogenetic analysis only isolates with good-quality sequences (Appendix Table 1). We included the DNA sequences of 1 isolate each of *R. microsporus* and *R. arrhizus* from our culture collection as controls. For DNA extraction, we used freshly grown sporulated culture suspended in 500 µL of lysis buffer and incubated for 5 minutes at 56°C. We then extracted DNA by using the phenol–chloroform–isoamyl alcohol (25:24:1) method, as previously described (*16*). We used PCR-based amplification of the 18S, internal transcribed spacer, and 28S genes, followed by sequencing using previously published primers for molecular identification (*17*). Because we obtained mixed chromatograms in most isolates of *R. homothallicus* by sequencing the internal transcribed spacer and 28S regions, we used the sequences of 18S for phylogenetic analysis. We aligned all sequences of the study isolates and reference sequences of various *Rhizopus* species obtained from GenBank by using MEGA 7.0 (*18*). We studied the evolutionary relationship of isolates by constructing a phylogenetic tree by using neighbor-joining analysis in MEGA 7.0. 

### AFST

We performed AFST of the isolates for amphotericin B, itraconazole, posaconazole, isavuconazole, and terbinafine in accordance with Clinical and Laboratory Standards Institute (CLSI) standards (*19*). In brief, we diluted the drugs in the standard Roswell Park Memorial Institute 1640 medium and then dispensed 100 μL into each well of 96-well microdilution trays. We tested MICs in the range of 0.03–16 μg/mL. We prepared the inoculum by harvesting the zygospores in 0.85% normal saline and adjusted spore counts by using a spectrophotometer at an absorbance of 530 nm. On adjusting the optical density (OD) to limits of 0.15–0.17 (0.4–5.0 × 10^4^ zygospores/mL), we observed no growth on subsequent incubation; hence, we used a higher OD of 0.2–0.3 (0.3–5.0 × 10^6^ zygospores/mL, confirmed by counting on hemocytometer). We diluted the suspensions to 1:50 in Roswell Park Memorial Institute 1640 medium and incubated the microtiter plates at 35°C for 4–8 days until visible growth was observed in the growth control. We defined MIC endpoints as the lowest drug concentration that inhibited any recognizable growth. We used *Candida krusei* (ATCC 6258) and *Aspergillus flavus* (ATCC 204304) as the quality-control strains.

Because of the absence or presence of fewer sporangiospores in *R. homothallicus*, performing AFST was challenging, and we modified the standard CLSI guidelines of AFST for this study. We used a higher OD with more inoculum containing both zygospores and hyphal fragments; performed intermittent vortexing of the inoculum for up to 45 minutes to ensure homogenous suspension (because zygospores tend to settle fast and are sometimes unevenly distributed between the wells); and conducted reading of AFST results up to 7 days (because zygospores take longer time to germinate or multiply compared with sporangiospores). The last step is in contrast to the standard protocol of reading the results at 24 hours for other medically important Mucorales, according to CLSI (*19*).

### AFLP Analysis 

We performed AFLP typing of *R. homothallicus* isolates as previously described (*20*). We used ≈50 ng of genomic DNA for the combined restriction–ligation procedure. We fragmented the DNA by using the 5 units each of restriction enzymes *Eco*RI and *Hin*dIII (New England Biolabs). For the preselective amplification, we used 10 μmol/L each of preselective primers of *Eco*RI primer (5′-GACTGCGTACCAATTC-3′) and *Hin*dIII (5′-GACTGCGTACCA GCTT-3′). We used the 6-carboxyfluorescein (6-FAM) labeled primers for selective amplification. We used 10 μmol/L each of *Hin*dIII primer (5′-ACTGCGTACCAGCTTT-3′) and *Eco*RI primer (5′-GACTGCGTACCAATTCAC-3′) for selective amplification. We performed capillary electrophoresis to identify the restricted DNA fragments and reference marker (LIZ 500) in a genetic analyzer (3500 Genetic Analyzer 8 Capillary Array; Applied Biosystems). We imported the fingerprint data into BioNumerics 6.6 (Applied Maths) and converted curves into bands for analysis. We calculated the genetic diversity by using the Pearson correlation coefficient and clustered the isolates by using the unweighted pair group method with arithmetic mean. Although no definite cutoff for differentiating between strains exists, we used an arbitrary cutoff of <70% for genus differentiation and >70% for species differentiation. We defined clonal isolates when the similarity was in the range of 95%–99%.

### Statistical Analysis

For statistical analysis, we used SPSS Statistics 22.0 (IBM) and GraphPad Prism 9.0 (GraphPad Software). We present the descriptive data as mean +SD for continuous variables and frequencies with percentages for categorical variables. We compared differences between the 2 groups by using the χ^2^ test and Fisher exact test for categorical variables, as appropriate, and used the Student *t*-test test to compare continuous data. We performed a binary logistic regression analysis of variables (age, sex, presence of brain involvement, combined medical–surgical therapy for ROM, and causative species [*R. homothallicus* vs. *R. arrhizus*]) that influenced the mortality rate for ROM and reported the adjusted odds ratio (aOR) and 95% CI. All the statistical tests were 2-sided, and we considered a p value <0.05 to be statistically significant.

## Results

Of the 631 patients with culture-confirmed mucormycosis, 43 (6.8%) had infection attributable to *R. homothallicus*. We excluded 324 cases from the study (262 because of inadequate information [e.g., lost to follow up], 35 because of non–*R. arrhizus* and non–*R. homothallicus* mucormycosis, and 27 non-ROM cases). We analyzed 41 ROM cases caused by *R. homothallicus* and 266 consecutive ROM cases attributable to *R. arrhizus*.

The mean +SD age of the 307 patients with ROM was 51.6 years +12.8 years, and most were men (205 [66.8%]). The mean age of patients with *R. arrhizus* infection was significantly higher than that for patients with ROM caused by *R. homothallicus*. Data on diabetes status were available for 279 patients; 93.9% had diabetes, and of those, 45 (16.1%) had diabetic ketoacidosis during the initial encounter. We observed no statistically significant difference between the risk factors proportion of patients with intracranial involvement or medical and surgical management in the 2 study groups (Table 2). The overall mortality rate was 35.2% (108/307) and was significantly higher among patients infected with *R. arrhizus* compared with *R. homothallicus* (39.1% vs. 9.8%; p = 0.0001). The mortality rate of patients with ROM caused by *R. homothallicus* was not significantly different between CAM and non-CAM subgroups (Appendix Table 2).

**Table 2:** Comparison of mucormycosis caused by *Rhizopus homothallicus* versus *R. arrhizus* in patients enrolled in a 10-month retrospective study, Chandigarh, India, January–October 2021*

Parameter	*R. homothallicus*, n = 41	*R. arrhizus*, n = 266	p value
Mean age, y (+ SD)	45.9 (± 12.8)	52.5 (± 12.7)	0.002
Sex			
M	23/41 (56.1)	182/266 (68.4)	0.15
F	18/41 (43.9)	84/266 (31.6)	
Risk factors			
CAM	23/41 (56.1)	256/266 (96.2)	0.0001
Duration after COVID-19, d (+ SD)	6.13 (± 13.5)	11.9 (± 14.8)	0.07
Diabetes mellitus	39/41 (95.1)	223/238 (93.7)	0.72
Recently diagnosed diabetes mellitus	9/39 (23.1)	54/223 (24.2)	
Renal transplantation	0	1/238 (0.4)	1.00
Intracranial involvement	3/41 (7.3)	13/266 (4.9)	0.46
Clinical features			
Fever	4/41 (9.8)	3/55 (5.5)	0.42
Headache	4/41 (9.8)	10/55 (18.2)	0.38
Toothache	4/41 (9.8)	5/55 (9.1)	0.91
Eye swelling	27/41 (65.9)	27/55 (49.1)	0.10
Facial pain	11/41 (26.8)	22/55 (40)	0.18
Facial swelling	16/41 (39)	22/55 (40)	0.92
Proptosis	3/41 (7.3)	0	0.08
Visual disturbance	18/41 (43.9)	7/55 (12.7)	0.0005
Oral ulcer	3/41 (7.3)	10/55 (18.2)	0.12
Nasal crust	5/41 (12.2)	5/55 (9.1)	0.62
Palatal eschar	5/41 (12.2)	10/55 (18.2)	0.42
Management			
Amphotericin therapy	38/41 (92.7)	212/218 (97.2)	0.14
LAMB	36/38 (94.7)	196/212 (92.5)	
Conventional AMB	2/38 (5.3)	16/212 (7.5)	
Surgery	24/36 (66.7)	184/245 (75.1)	0.31
30-day mortality	4/41 (9.8)	104/266 (39.1)	0.0001

*Values are no. patients/no. with data available (%) except as indicated. CAM, COVID-19–associated mucormycosis; LAMB, liposomal amphotericin B; AMB, amphotericin.

On multivariate analysis, infection with *R. homothallicus* and surgery for ROM were independently associated with lower odds of death. A higher age (odds ratio [OR] 1.01 [95% CI 1.03–1.08]) and the presence of brain involvement (OR 22.7 [95% CI 4.03–128.10]) were independently associated with higher mortality rates among ROM cases (Table 3).

**Table 3:** Binary logistic regression analysis demonstrating the factors associated with death among patients with rhino-orbital mucormycosis enrolled in a 10-month retrospective study, Chandigarh, India, January–October 2021*

Variable	Survivors	Nonsurvivors	Odds ratio (95% CI)	p value
Mean age, y (+ SD)	55.8 (+ 12.4)	49.4 (+ 12.6)	1.06 (1.03–1.08)	0.0001
Male sex	124/199 (62.3)	81/108 (75)	1.38 (0.75–2.53)	0.31
Intracranial involvement	4/199 (2)	12/108 (11.1)	22.7 (4.03–128.1)	0.0001
Surgery for ROM	144/173 (83.2)	64/108 (59.3)	0.22 (0.11–0.43)	0.0001
*R. homothallicus* infection	37/199 (18.6)	4/108 (3.7)	0.08 (0.02–0.36)	0.001

*Values are no. patients/no. with data available (%) except as indicated. ROM, rhino-orbital mucormycosis.

We performed AFST for 34 *R. homothallicus* isolates. We calculated the geometric mean, range, and MICs at which 50% (MIC_50_) and 90% (MIC_90_) of isolates are inhibited for amphotericin B, itraconazole, posaconazole, isavuconazole, and terbinafine (Table 4). 

**Table 4:** Distribution of MICs of 34 *Rhizopus homothallicus* isolates from patients enrolled in a 10-month retrospective study, Chandigarh, India, January–October 2021*

Antifungal agent	Geometric mean (range), mg/L	MIC_50_, mg/L	MIC_90,_ mg/L
Amphotericin B	0.75 (0.03–16)	2	4
Itraconazole	0.51 (0.03–16)	0.5	8
Posaconazole	0.24 (0.03–8)	0.12	2
Isavuconazole	0.32 (0.03–16)	0.25	2
Terbinafine	0.34 (0.03–16)	0.25	4

*MIC_50_, MIC at which 50% of isolates are inhibited; MIC_90_, MIC at which 90% of isolates are inhibited.

**Figure 2:**
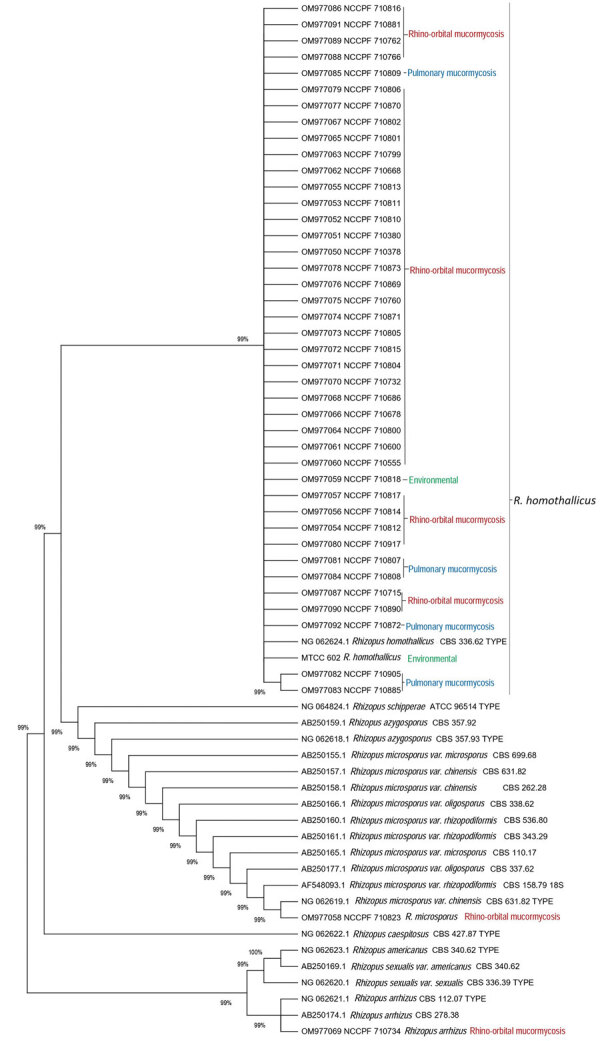
Evolutionary relationships of 40 *Rhizopus homothallicus* isolates from patients in a 10-month study, Chandigarh, India, January–October 2021, and 2 environmental isolates. GenBank accession numbers of 41 clinical and 1 environmental–MTCC 602 isolate of *R. homothallicus* from India are shown. Tree generated using neighbor-joining algorithm with 1,000 bootstrap replicates.

The phylogram constructed using 18S sequences (40 clinical and 2 environmental of *R. homothallicus* and 1 each of *R. arrhizus* and *R. microsporus*) (Figure 2; Appendix Table 1) and the AFLP results (Appendix Figure) both revealed distinct clustering of *R. homothallicus* from the other tested species. We observed no prominent clades among isolates of *R. homothallicus* from patients with CAM versus non-CAM.

## Discussion

We report a 6.8% prevalence of *R. homothallicus* infection among culture-confirmed mucormycosis cases. The mortality rate for ROM caused by *R. homothallicus* was significantly less than that for *R. arrhizus* (9.8% vs. 39.1%) after adjusting for age, sex, intracranial extension of the disease, and surgery for mucormycosis in the binary logistic regression model. The AFST data indicated good susceptibility to the common antifungal drugs and the newer agent isavuconazole. The 18S gene sequencing and AFLP revealed distinct clustering of *R. homothallicus* from the common species implicated in ROM.

Human infection with *R. homothallicus* was first reported from North India (*4*) in a case of pulmonary mucormycosis and was followed by a few more reports (*5*,*21*). In 2 prospective multicenter studies from India, *R. homothallicus* accounted for 2.5% (6/239) (*22*) and 7.6% (22/290) (*23*) of the cases where the causative organism could be identified. Despite a higher prevalance of mucormycosis cases (associated with the CAM outbreak) in our study, the prevalence of *R. homothallicus* mucormycosis (6.8%) was similar that in the previous reports.

*R. homothallicus*, a homothallic fungus, produces more heavy sexual spores than asexual spores (sporangiospores) and undergoes less dispersion in the air. The relative amount of sporangiospores is considerably lower in *R. homothallicus* than in the heterothallic *Rhizopus* species (*24*). Consequently, the chances of acquiring infection with *R. homothallicus* are expected to be low. Thus, *R. homothallicus* infections probably indicate the presence of some unidentified environmental niche where this agent can produce asexual spores abundantly and disperse them in the air. Recent studies have shown *R. homothallicus* in the hospital air and air samples obtained from the residence of patients with mucormycosis (*25*,*26*).

The mortality rate for mucormycosis may vary with the causative species. For instance, 2 systematic reviews have shown higher mortality rates for infections with *Cunninghamella* spp. than those with *R. arrhizus* (*2*,*27*). We observed a significantly better survival rate with *R. homothallicus* infection compared with *R. arrhizus*. A substantially higher proportion of patients with *R. homothallicus* infection had visual disturbance (44% vs. 13% for *R. arrhizus*), and this difference could have led to earlier detection. The duration of symptoms in CAM patients with mucormycosis caused by *R. homothallicus* and *R. arrhizus* was also different, although not significantly (6 vs. 12 days; p = 0.07). The timely initiation of antifungal therapy, intracranial spread of disease, and surgery for mucormycosis are important factors determining the outcome of mucormycosis (*28*,*29*). We conducted a preliminary growth curve analysis at 25°C, 37°C, and 40°C (data not shown) to determine whether the 2 species had a difference in growth rate. We observed an overall faster time to log phase at all temperatures for *R. arrhizus* compared with *R. homothallicus* (6 h vs. 27 h at 25°C, 5 h vs. 32 h at 37°C, and 6 h vs. 25 h at 40°C). However, further in vivo studies are required to identify the different pathogenic potentials of these 2 Mucorales species.

The first limitation of our study was that it was retrospective and conducted at a single center, limiting generalizability. We do not have information on all patients with ROM caused by *R. arrhizus*, and the proportion of patients with CAM was much higher in the *R. arrhizus* group than in the *R homothallicus* group. However, data from our center and another large multicenter study from India showed similar mortality rates for ROM with or without COVID-19 co-infection (*8*,*11*). Because our study focused exclusively on ROM cases, future studies should explore pulmonary or other sites of involvement, which are inherently associated with higher mortality rates (*30*). Although we noted a lower mortality rate from *R. homothallicus* infection on multivariate analysis, we cannot exclude residual confounding factors that could have resulted in improved survival with *R. homothallicus* infection. Also, we do not have a detailed evaluation of risk factors or genetic analysis to ascertain whether specific factors predispose persons to infection with *R. homothallicus*. Alhough the AFLP method is known for poor reproducibility, the results may be within an acceptable range when the test is repeated with the same batch of reagents, as we observed with our *R. homothallicus* isolates*.* Moreover, modification of the AFST protocol makes the results difficult to interpret and compare with published data.

In conclusion, our results show that *R. homothallicus* is an important agent of mucormycosis with epidemiologic and clinical significance. *R. homothallicus* may be less virulent or manifest earlier than *R. arrhizus*, thus resulting in better survival. Identifying *R. homothallicus* based on macroscopic and microscopic appearance is not difficult and should be emphasized. Most *R. homothallicus* isolates are uniformly susceptible to the commonly used antifungal agents to manage mucormycosis.

Appendix Additional information about clinical and mycologic characteristics of emerging mucormycosis agent *Rhizopus homothallicus*.
