# Supplementary material for: Clinical and Mycologic Characteristics of Emerging Mucormycosis Agent Rhizopus homothallicus
Source: Emerg Infect Dis. 2023 Jul;29(7):1313–22. doi: 10.3201/eid2907.221491 (PMC10310386; doi:10.3201/eid2907.221491)
Supplement: Appendix — Additional information about clinical and mycologic characteristics of emerging mucormycosis agent Rhizopus homothallicus. [file 22-1491-Techapp-s1.pdf]

Article DOI: https://doi.org/10.3201/eid2907.221491

Clinical and Mycologic Characteristics of Emerging Mucormycosis Agent *Rhizopus homothallicus*

Appendix

Appendix Table 1. Accession numbers of 18 S ribosomal RNA sequences of the isolates used in the present study.

S no.	NCCPF no.	NCBI accession no.	Species	Site of isolation
1	710378	OM977050	*R. homothallicus*	Rhino-orbital mucormycosis
2	710380	OM977051	*R. homothallicus*	Rhino-orbital mucormycosis
3	710810	OM977052	*R. homothallicus*	Rhino-orbital mucormycosis
4	710811	OM977053	*R. homothallicus*	Rhino-orbital mucormycosis
5	710812	OM977054	*R. homothallicus*	Rhino-orbital mucormycosis
6	710813	OM977055	*R. homothallicus*	Rhino-orbital mucormycosis
7	710814	OM977056	*R. homothallicus*	Rhino-orbital mucormycosis
8	710817	OM977057	*R. homothallicus*	Rhino-orbital mucormycosis
9	710823	OM977058	*R. microsporus*	Rhino-orbital mucormycosis
10	710818	OM977059	*R. homothallicus*	Environmental
11	710555	OM977060	*R. homothallicus*	Rhino-orbital mucormycosis
12	710600	OM977061	*R. homothallicus*	Rhino-orbital mucormycosis
13	710668	OM977062	*R. homothallicus*	Rhino-orbital mucormycosis
14	710799	OM977063	*R. homothallicus*	Rhino-orbital mucormycosis
15	710800	OM977064	*R. homothallicus*	Rhino-orbital mucormycosis
16	710801	OM977065	*R. homothallicus*	Rhino-orbital mucormycosis
17	710678	OM977066	*R. homothallicus*	Rhino-orbital mucormycosis
18	710802	OM977067	*R. homothallicus*	Rhino-orbital mucormycosis
19	710686	OM977068	*R. homothallicus*	Rhino-orbital mucormycosis
20	710734	OM977069	*R. arrhizus*	Rhino-orbital mucormycosis
21	710732	OM977070	*R. homothallicus*	Rhino-orbital mucormycosis
22	710804	OM977071	*R. homothallicus*	Rhino-orbital mucormycosis
23	710815	OM977072	*R. homothallicus*	Rhino-orbital mucormycosis
24	710805	OM977073	*R. homothallicus*	Rhino-orbital mucormycosis
25	710871	OM977074	*R. homothallicus*	Rhino-orbital mucormycosis
26	710760	OM977075	*R. homothallicus*	Rhino-orbital mucormycosis
27	710869	OM977076	*R. homothallicus*	Rhino-orbital mucormycosis
28	710870	OM977077	*R. homothallicus*	Rhino-orbital mucormycosis
29	710873	OM977078	*R. homothallicus*	Rhino-orbital mucormycosis
30	710806	OM977079	*R. homothallicus*	Rhino-orbital mucormycosis
31	710917	OM977080	*R. homothallicus*	Rhino-orbital mucormycosis
32	710807	OM977081	*R. homothallicus*	Pulmonary mucormycosis
33	710905	OM977082	*R. homothallicus*	Pulmonary mucormycosis
34	710885	OM977083	*R. homothallicus*	Pulmonary mucormycosis
35	710808	OM977084	*R. homothallicus*	Pulmonary mucormycosis
36	710809	OM977085	*R. homothallicus*	Pulmonary mucormycosis
37	710816	OM977086	*R. homothallicus*	Rhino-orbital mucormycosis
38	710715	OM977087	*R. homothallicus*	Rhino-orbital mucormycosis
39	710766	OM977088	*R. homothallicus*	Rhino-orbital mucormycosis
40	710762	OM977089	*R. homothallicus*	Rhino-orbital mucormycosis
41	710890	OM977090	*R. homothallicus*	Rhino-orbital mucormycosis
42	710881	OM977091	*R. homothallicus*	Rhino-orbital mucormycosis
43	710872	OM977092	*R. homothallicus*	Pulmonary mucormycosis

NCCPF National Culture Collection of Pathogenic Fungi, NCBI National Center for Biotechnology Information.

Appendix Table 2. Comparison of various clinical and demographic parameters between patients with CAM and Non-CAM due to *R. homothallicus.*

Parameter	*R. homothallicus* CAM n-23(%)	*R. homothallicus* Non-CAM n-18 (%)	P value
Age (years)	46.6 ± 12.7	44.8 ± 13.1	0.667
Gender (F)	12 (52.2)	6 (33.3)	0.343
DM	21 (91.3)	18 (100)	0.495
UDM	20 (95.2)	10 (55.6)	0.006
Recently diagnosed diabetes	8 (38.1)	1 (5.6%)	0.023
Hypertension	7 (30.4)	1 (5.6)	0.059
Steroid	6 (26.1)	0 (0)	0.027
Clinical features			
Duration of symptom at presentation (days)	4.09 ± 1.4	7.9 ± 8.5	0.040
Fever	0	4 (22.2)	0.030
Headache	2 (8.7)	2 (11.1)	1.000
Toothache	4 (17.4)	0	0.118
Eye swelling	20 (87)	7 (38.9)	0.002
Facial pain	5 (21.7)	6 (33.3)	0.489
Facial swelling	10 (43.5)	6 (33.3)	0.540
Proptosis	0	3 (16.7)	0.077
Visual disturbance	8 (34.8)	10 (50)	0.219
Oral ulcer	3 (13)	0	0.243
Nasal crust	3 (13)	2 (11.1)	1.000
Palatal eschar	2 (8.7)	3 (16.7)	0.638
Treatment/Outcome			
30 d mortality	1 (4.3)	3 (16.7)	0.303
Antifungal	22 (95.7)	16 (88.9)	0.573
LAMB	22 (95.7)	14 (77.8)	0.150

CAM: COVID-19 associated mucormycosis; DM- Diabetes mellitus; UDM – uncontrolled diabetes mellitus; LAMB- liposomal amphotericin B.

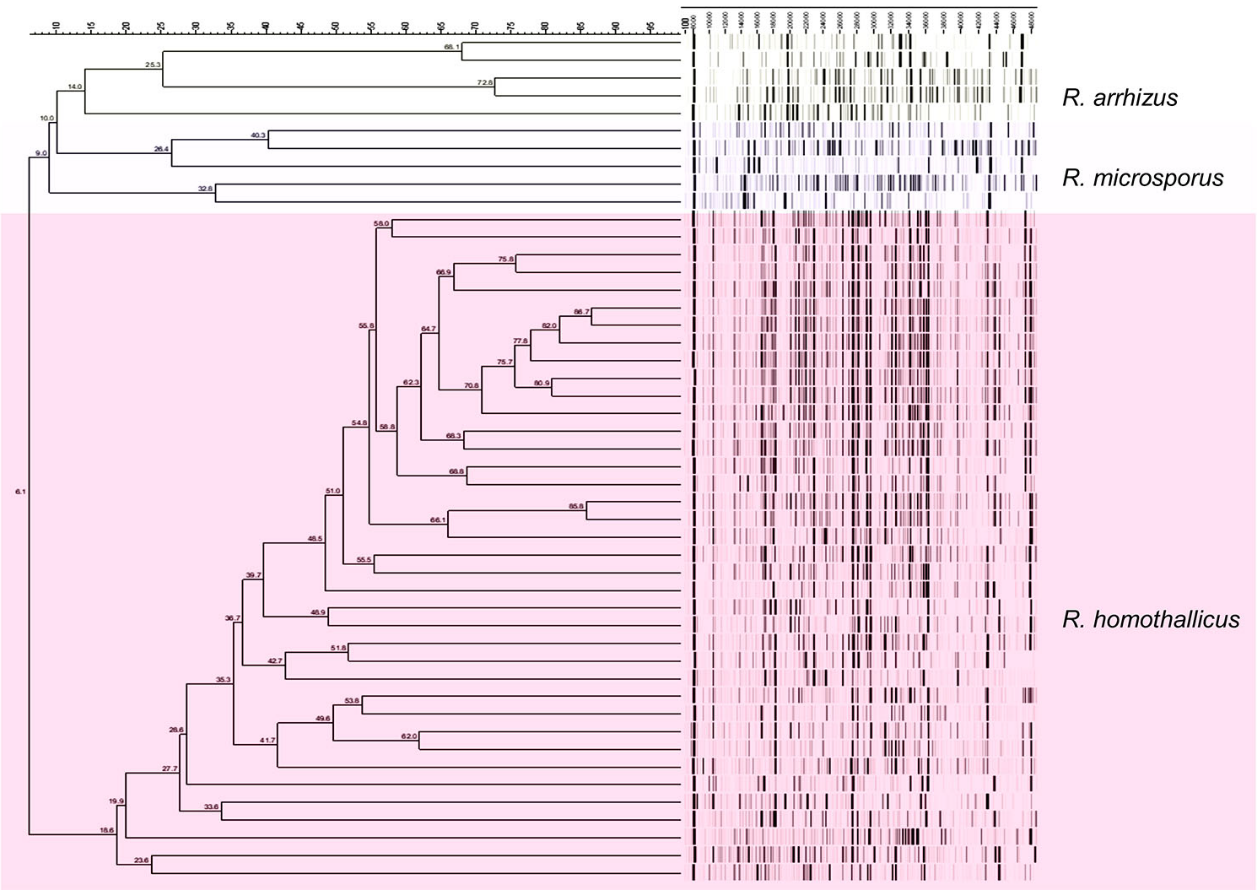

Appendix Figure. Amplified fragment length polymorphism analysis showing fingerprints of common species of genus *Rhizopus*. The range of fragment lengths was between 100–480 bp, as denoted on the scale bar (top).
